# Detection of EpCAM-Negative and Cytokeratin-Negative Circulating Tumor Cells in Peripheral Blood

**DOI:** 10.1155/2011/252361

**Published:** 2011-04-19

**Authors:** Stephen D. Mikolajczyk, Lisa S. Millar, Pavel Tsinberg, Stephen M. Coutts, Maryam Zomorrodi, Tam Pham, Farideh Z. Bischoff, Tony J. Pircher

**Affiliations:** ^1^Research and Development, Biocept Inc., 5810 Nancy Ridge Drive, Suite 150, San Diego, CA 92121, USA; ^2^Translational and Clinical Development, Biocept Inc., 5810 Nancy Ridge Drive, Suite 150, San Diego, CA 92121, USA

## Abstract

Enrichment of rare circulating tumor cells (CTCs) in blood is typically achieved using antibodies to epithelial cell adhesion molecule (EpCAM), with detection using cytokeratin (CK) antibodies. However, EpCAM and CK are not expressed in some tumors and can be downregulated during epithelial-to-mesenchymal transition. A micro-fluidic system, not limited to EpCAM or CK, was developed to use multiple antibodies for capture followed by detection using CEE-Enhanced (CE), a novel *in situ* staining method that fluorescently labels the capture antibodies bound to CTCs. Higher recovery of CTCs was demonstrated using antibody mixtures compared to anti-EpCAM. In addition, CK-positive breast cancer cells were found in 15 of 24 samples (63%; range 1–60 CTCs), while all samples contained additional CE-positive cells (range 1–41; median = 11; *P* = .02). Thus, antibody mixtures against a range of cell surface antigens enables capture of more CTCs than anti-EpCAM alone and CE staining enables the detection of CK-negative CTCs.

## 1. Introduction

In order to analyze rare CTCs in the blood of cancer patients, it is necessary to enrich, isolate and identify the tumor cells in the presence of billions of red blood cells and the tens of millions of nucleated hematopoietic cells. The most commonly used form of enrichment relies on antibodies against the epithelial cell adhesion molecule, EpCAM [1, 2]. The FDA-approved CellSearch system has set the standard for the use of EpCAM in the enrichment of CTCs using a magnetic ferrofluid approach [3, 4]. EpCAM is also used as a main capture component in other immunomagnetic bead-based systems as well as microfluidic systems [5–7]. Other emerging approaches do not depend on immuoenrichment at all but instead use precise size filters to separate larger epithelial cells from smaller red blood cells (RBCs) and white blood cells (WBCs) [8]. Alternatively, approaches using only cell lysis to remove interfering RBC have been described [9, 10]. In this case all remaining nucleated cells remaining after RBC lysis are layered onto several slides for further analysis. Systems using PCR for detection do not enumerate based on visual cell detection, but still use immunomagnetic beads coated with anti-EpCAM antibodies, among others, for enrichment [7, 11, 12].

Regardless of the system used for isolation or enrichment, detection almost always relies on staining for cells containing cytokeratin, an internal architectural protein that is largely associated with epithelial cells [13]. Most healthy control blood contains few or no CK-positive cells [3]. Counterstaining with anti-CD45 is employed to rule out occasional nucleated WBC that stain for CK. In those cases where EpCAM has not been used for enrichment, such as the RBC lysis approach, EpCAM can alternatively be used for detection [10, 14]. PCR-based approaches generally use some combination of anti-EpCAM or anti-CK for enrichment or detection before DNA is extracted for analysis [12, 15, 16]. Thus, there is a high dependence on just two epithelial markers for capture and/or detection of CTCs.

Using the above criteria, it is implicitly understood that the detection of CTCs is actually the detection of circulating epithelial cells that are not typically present in blood, but which can be detected as tumor-derived cells in the blood of cancer patients. It has become axiomatic in the field that all CK and/or EPCAM positive, CD45-negative cells with a nucleus in cancer patients are CTCs. A number of studies using CellSearch have shown a good correlation between the numbers of these circulating CK-positive/EpCAM-positive cells and prognosis for cancer survival [17, 18]. There is also considerable evidence that some of the CK-positive cells contain cancer cytogenetic markers such as TMPRSS2-ERG, MYC, PTEN, and Her2/neu [19–22]. The success in correlating CTC enumeration with patient survival has conferred a dependence on EpCAM and CK to virtually every other system. This has also imposed a clear bias on the study of CTCs, primarily the failure to include tumor cells that have reduced or absent CK and/or EpCAM. The failure to identify such cells limits investigations into additional tumor types.

EpCAM is expressed in most but not all tumors [23]. There is evidence for upregulation and down-regulation of EpCAM with cancer progression and metastasis, and it is likely that both are true, depending on the type and stage of cancer and other biological variables not yet well understood [24, 25]. CK is heterogeneously expressed in tumor, and may be downregulated or secreted [26, 27]. During the progression of epithelial-to-mesenchymal transition (EMT) both EpCAM and CK are downregulated as part of an oncogenic pathway to increased invasiveness and metastatic potential [2]. EpCAM may be downregulated to allow epithelial cell dissociation from the tumor, and the structural cytoplasmic CK is downregulated to facilitate cell plasticity and migration. 

Given the potential range of genotypic etiology it may be difficult or impossible to predict the predominant phenotype of any given CTC in a sample. Significant phenotypic heterogeneity may exist between samples, or even among the cells in a single sample. And yet the field has been slow to progress beyond the simple EpCAM capture, CK detection model because there is no clear alternative. In order to extend the range of possible tumor cell enrichment it is necessary to have a system with greater flexibility to enrich and detect additional types of CTCs.

In this study we report a platform that can be used to simultaneously capture multiple circulating tumor cell types by employing mixtures of antibodies that may include, but are not solely dependent on, EpCAM. In addition, we describe a novel universal staining method, CEE-Enhanced that can selectively detect tumor cells that have been targeted by the capture antibodies, regardless of their phenotypic expression of other known cancer markers.

## 2. Materials and Methods

### 2.1. Blood Collection

Blood samples from cancer patients were obtained from Conversant Biologics Inc., Huntsville, Ala. All samples were collected using an IRB approved protocol and informed consent. As controls, healthy donors who had no history of cancer also provided informed consent prior to participation. Blood samples were collected into 10-mL Vacutainer tubes containing 1.5 mL acid-citrate-dextrose (ACD Solution A Vacutainers; Becton, Dickinson and Company, Franklin Lakes, NJ.). Within 60 minutes of blood collection, the addition of 250 *μ*L of anticlumping reagent (CEE-Sure; Biocept, San Diego, Calif.) was injected into each tube before being shipped to Biocept for processing within 24 hours of collection. Samples were stored at room temperature (RT) before processing.

### 2.2. Blood Sample Processing

Blood samples were initially processed for recovery of peripheral blood mononuclear cells by using a Percoll density gradient method and Leucosep tubes (Greiner Bio-One, Monroe, NC.). Each Leucosep tube was prefilled with Percoll Plus (GE Healthcare, Piscataway, NJ.) at a density of 1.083 g/mL (adjusted using normal saline) and stored at RT. Each 10-mL blood sample was diluted threefold with phosphate-buffered saline (PBS) containing 1 mg/mL casein and 1 mM ethylenediaminetetraacetic acid (EDTA) and poured directly into a Leucosep tube. Samples were centrifuged for 15 minutes at 1000× g at RT in swinging bucket rotors (Allegra X-12R centrifuge; Beckman Coulter, Brea, Calif.), with brakes set to their lowest setting. After centrifugation, the upper layer (above the separation barrier) was decanted through a 70-*μ*m cell strainer into a 50-mL conical tube. The decanted sample volume was adjusted to 45 mL with PBS/casein/EDTA and then centrifuged for 10 minutes at 400× g. Supernatant was removed by aspiration. The pellet was then resuspended and incubated with Fc blocker (100 *μ*g/mL human IgG) and capture antibody cocktail (each antibody adjusted to 1 *μ*g/mL) for 30 minutes at RT. After incubation, the pellet was washed by adjusting the volume to 45 mL with PBS/casein/EDTA and centrifuging for 10 minutes at 400× g at RT. Biotinylated anti-mouse secondary (Jackson Labs, Bar Harbor, Me) was added to the pellet and after mixing, was incubated for 30 minutes at RT. The resulting pellet was washed three times with PBS/casein/EDTA. Each wash step consisted of centrifugation for 10 minutes at 400× g, followed by supernatant aspiration. The final pellet was suspended in 1 mL PBS/BSA/EDTA and subjected to capture and staining on the Cell Enrichment and Extraction (CEE) microchannel (manufactured at Biocept, Inc., San Diego, Calif.). Samples were pulled through CEE microchannels with syringe pumps (manufactured at Biocept Inc., San Diego, Calif.) connected to the outlet at a volumetric flow rate of 18 *μ*L/min. After the entire sample was processed through the channel, cells were cross-linked within CEE microchannels with 2 mM NHS homobifunctional protein cross-linker and fixed with 80% MeOH.

### 2.3. Antibody Mixture

Unless otherwise specified the capture antibody mixture contained the following individual antibodies: anti-EpCAM (Trop-1), tumor-associated calcium signal transducer 2 (Trop-2), (BD Biosciences, San Diego, Calif.); anti-c-MET (95106), anti-Folate-binding protein receptor (MOV18), (R&D Systems, Minneapolis, Minn.); anti-N-Cadherin (GC-4), (Sigma-Aldrich, St. Louis, Mo.); anti-CD318 (CUB1), antimesenchymal stem cell antigen (W305), anti-Her2 (24D2), (Biolegend, San Diego, Calif.); anti-MUC-1 (M4H2) (Fitzgerald, Acton, Mass); and anti-EGFR (528) (Santa Cruz Biotechnology, Santa Cruz, Calif). Each antibody was assessed by flow cytometry (Accuri Cytometers Inc., Ann Arbor, Mich) for positive signal on multiple cultured cell lines, for example, SKOV (ovarian), SKBR3 (breast), T24 (bladder), LNCaP (prostate), and for low background on buffy coat cells isolated from control blood.

### 2.4. Microchannel and Detection of Captured CTCs on the Microchannel

**Figure 1:**
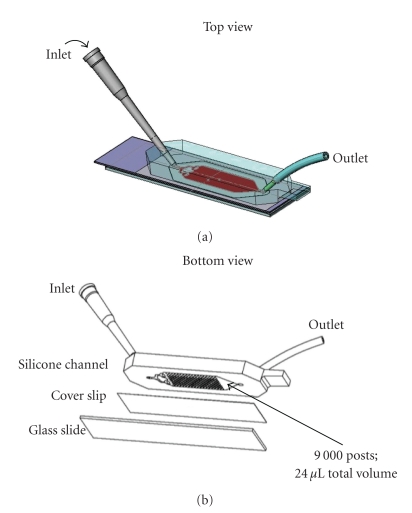
Diagram of the CEE microchannel. (a) Top view of the channel showing the inlet where sample is loaded and the outlet that is attached to a syringe pump to draw sample through the channel. (b) Bottom view shows the area where 9,000 posts are located in the silicone block and the channel sealed with the bottom cover slip. The total volume of the microchannel is 24 *μ*L. A standard microscope slide is added for stability during handling but is removed to visualize cells. The microchannel is inverted on a microscope and the captured cells viewed through the coverslip.

CEE microchannel design is illustrated in Figure 1. The random size and spacing of the posts is mathematically designed to avoid laminar flow through the channel, thus maximizing cell contact with the inner surfaces. The entire inner surface of the channel is derivatized with tethered streptavidin and therefore cells may be specifically bound on any surface. In practice the majority of the CTCs are captured on the posts though some cells are found on the channel floor.

Cells were stained with a mixture of anticytokeratin 7/17 (clone C-46), 18 (clone DA/7), 19 (clone A53-B/A2), and pan-cytokeratin (clone C-11) (BioLegend, San Diego, Calif.) antibodies labeled with AlexaFluor-488; CD45 antibody (clone HI30) (BioLegend, San Diego, Calif.) labeled with AlexaFluor-594 for 30 minutes, washed with PBS and stained with DAPI III to visualize the nucleus. Channels were stored at +8°C until microscopic analysis. CTC enumeration was performed by analysis through a standard Olympus BX51 fluorescence microscope (Olympus America Inc., Center Valley, Pa.) at 200 X magnifications and based on CK+/CD45-/DAPI+ stain criteria. The precise location (X- and Y-stage coordinates) of each CTC was recorded, permitting relocalization of cells after additional staining procedures.

CE staining employed *in situ* labeling of captured cells on the microchannel using 5 *μ*g/mL neutravidin labeled with fluorescent probes, including AlexaFluor 488 or 546 as indicated. Following initial scoring and localization of CK+/CD45−/DAPI+ cells, the captured cells within the microchannel were then subjected to CE staining whereupon the channels were rescored to note the locations of the CE-labeled cells as well as to assess presence of CE-stain on the original CK stained cells.

### 2.5. Fluorescence *in situ* Hybridization (FISH)

Following CTC enumeration of the breast cancer samples in Figure 7, the CEE microchannels were processed for multi-color FISH using the FDA approved PathVysion HER-2 DNA Probe Kit (centromere 17 specific probe (CEP 17-Spectrum Green) and locus-specific HER2 probe (Spectrum orange)) and a centromere-specific probe to chromosome 8 (CEP 8-Spectrum Aqua, Abbott Molecular). Each of the microchannels was first dehydrated before the addition of the probe mixture. Codenaturation of the probe mixture was performed on a ThermoBrite unit (Abbott Laboratories) at 95°C followed by hybridization at 37°C overnight. Postwash was performed at 74°C in 0.4× saline-sodium citrate (SSC) buffer containing 0.3% IPEGAL (Sigma-Aldrich, St. Louis, MO.) followed by 2× SCC wash containing 0.1% IPEGAL and then DAPI (blue). The CEE channels were imaged on the Olympus BX51 fluorescence microscope equipped with filters to view DAPI, SpectrumAqua, SpectrumOrange, and SpectrumGreen (Olympus America Inc.). Images were analyzed with use of the ISIS imaging system v5.2 (Metasystems, Waltham, Mass.). Evaluation of FISH signal patterns was performed on both CK-positive and CE-positive cells in the microchannel. CTCs were identified. The ratio of HER2 : CEP 17 was calculated and a ratio >2.2 was regarded as positive for HER2 gene amplification.

## 3. Results

### 3.1. Microchannel Capture Efficiency Using Multiple Antibodies

**Figure 2:**
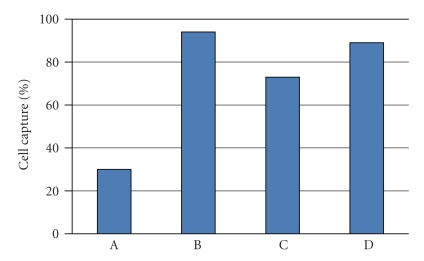
Capture of T24 and SKOV cells spiked into blood. A: the percentage capture of T24 cells using anti-EpCAM antibody. B: T24 capture % using anti-EpCAM and TROP-2 antibodies. C: SKOV capture % using anti-EpCAM antibody; D: SKOV capture % using anti-EpCAM and TROP-2 antibodies. By FACS T24 cells were shown to contain 4,000 and 60,000 EpCAM and TROP-2 antigens, respectively; SKOV cells were shown to contain 66.000 and 12,000 EpCAM and TROP-2 antigens, respectively. Antibody capture is less efficient with low-antigen expression on the cells, but increases in an additive manner when antibodies are used in combination.

The efficiency of cell capture using immuno-based systems is dependent on the presence and number of surface antigens on the cells.  Figure 2 shows the difference in capture using T24 and SKOV cell lines when incubated with either anti-EpCAM only and with a mixture of 2 antibodies, anti-EpCAM and Trop-2. As determined by flow cytometry, T24 and SKOV cells had about 4,000 and 60,000 EpCAM antigens, respectively, and the inverse level of Trop-2 antigens, about 60,000 and 12,000. The recovery of T24 cells increases from 30% with anti-EpCAM only, to 90% when the cells were incubated with both anti-EpCAM and Trop-2. SKOV cells were recovered at 80% with anti-EpCAM and only marginally higher when Trop-2 was added. Since antibodies in the current system serve to populate the surface of the cells with biotin, these results demonstrate that antibodies bound to the surface of the cells are additive with respect to capture on the microchannel, and are not mutually exclusive. 

**Figure 3:**
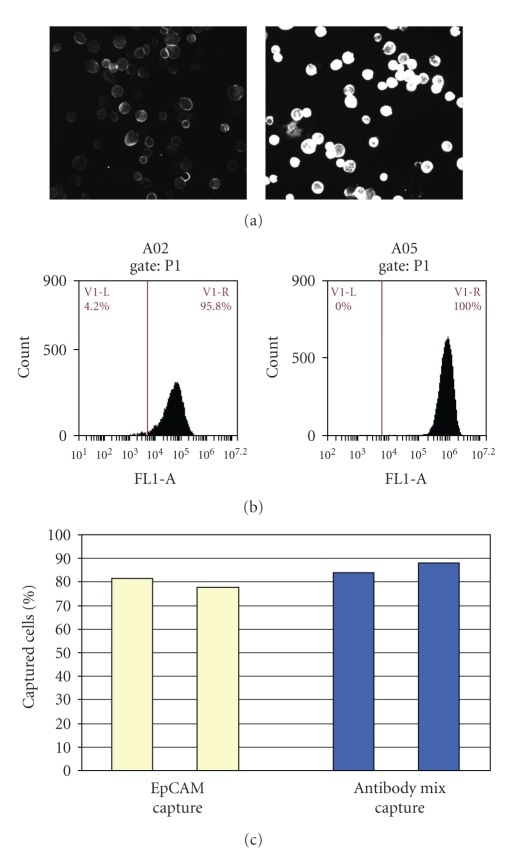
Capture and stain of SKOV cells with EpCAM and with an antibody mixture. (a) The relative stain intensity of these cells on a microscope slide using anti-mouse-AlexaFluor488 when the cells were preincubated with anti-EpCAM antibody only, or with an antibody mixture of anti-HER2/neu, anti-CD44 and anti-CD28. (b) The FACS profile of the respective antigens of EpCAM or of the 3-antibody mixture present on each cell. (c) The percentage capture of SKOV cells when preincubated with anti-EpCAM or the antibody mixture. This shows that much lower antigen levels are necessary for good cell capture than for good staining intensity. Antibody mixtures improve CE staining efficiency.

Additional studies on SKOV cells further demonstrate that while anti-EpCAM alone may be sufficient for good capture, additional antibodies improve detection when staining with CE.  Figure 3 shows the capture of SKOV cells with anti-EpCAM only and with a small antibody mixture containing anti-Her2/neu, anti-CD44 and anti-CD26. Flow cytometry showed an average of 66,000 EpCAM antigens and 620,000 surface antigens using the mixture (Figure 3(b)). There is no significant difference in the cell capture with anti-EpCAM alone or with the antibody mixture (Figure 3(c)). However, when SKOV cells incubated with anti-EpCAM-only were stained using the CE protocol to label surface antibodies, there was only faint staining, while the same cells with multiply bound antigens had significantly higher stain intensity as shown when the cells were viewed on microscope slides (Figure 3(a)). This demonstrates that a mixture of antibodies may not be needed to capture a given cell but the extra antibody density on the surface of the cell significantly improves the staining intensity of the cell when using CE. Cells on the microchannel were similarly detected using either anti-CK or the CE protocol alone (discussed below). 

### 3.2. Capture and Detection of CTCs

The immunostaining intensity by CK or CE is based on the number of antigens. 

**Figure 4:**
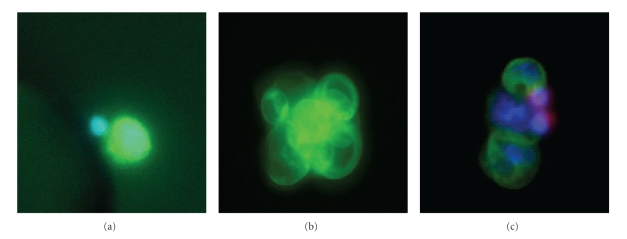
Immunofluorescent staining. (a) A LnCAP cell spiked into blood and captured on the microchannel, stained for CK (green) and also nuclear stained with DAPI (blue). A small WBC is seen with only the nucleus stained blue. (b) A cluster of CTCs from a clinical lung cancer sample captured on the microchannel that are stained for CK. These cells were CD45-negative and DAPI-positive (not shown). (c) A cluster of cells from lung cancer showing triple staining with CK (green), CD45 (red), and DAPI (blue). Three CK-positive CTCs are shown with 2 smaller WBCs stained positive for CD45.

**Figure 5:**
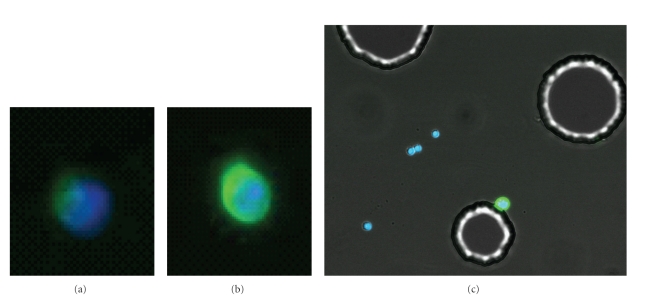
The use of CEE-Enhanced to improve detection of cells on the microchannel. (a) A clinical breast cancer CTC stained for CK and nuclear-stained with DAPI. This cell is weakly CK positive. (b) The same cell after subsequent stain with CE labeled with the same AlexaFLuor-488 fluorophore in order to enhance the stain intensity. (c) SKOV cell spiked into blood and recovered on the microchannel using an antibody mixture (see Section 2). Cells on the channels were stained with CE-488 and DAPI. The four WBCs stained blue for DAPI only, while the SKOV (attached to post) can be detected only with CE (green). This higher contrast image shows the outline of the posts in the microchannel. Together these images show that CE can be used to augment weakly staining CK cells or can be used to detect cells without CK stain.

For cell lines and many of the CTCs visualized in most systems, CK staining is clearly visible by manual microscopy. Single cells as well as small microemboli may be captured in the microchannel (Figures 4(a)–4(c)) since the spacing between the posts is sufficient to allow larger clumps of cells to pass between the posts on the channel. However there is a gradient of staining intensities in CTCs such that some are not easily detectable above background. In those cases where there is weak CK staining in a CD45-negative cell, it may still be difficult to distinguish a true positive from background. The clinical example of this is seen in Figure 5(a) which shows a CTC captured from a breast cancer sample that was weakly CK positive. The location for this cell on the microchannel was recorded and the cell was relocated after subsequent staining with CE. In Figure 5(b) the same cell was more intensely stained after treatment with CE. This demonstrates that the cell was clearly captured due to multiple capture antibodies on its surface, but the endogenous CK itself was low or downregulated. Increased staining intensity was routinely observed on weakly CK positive cells.  Figure 5(c) shows an SKOV cell spiked into blood and captured on the microchannel with the antibody mixture. CE only was used to detect this cell. The high contrast image also shows the outline of the posts on the channel. Background WBCs remain DAPI positive only. Figure 5 shows that CTCs may be stained *de novo* with CE, and that CE can also be used to enhance weakly stained CK-positive cells. 

Table 1 shows the comparison between capture with anti-EpCAM alone and with an antibody mixture, as detected with anti-CK stain. Duplicate tubes of blood from metastatic cancer patients were incubated with anti-EpCAM only and with the antibody mixture. On an individual basis, the effect of using an antibody mixture can range from no increase in CK-positive cells to severalfold higher. Overall, the antibody mixture captured significantly more CK-positive cells than anti-EpCAM alone (mean 18.5 versus 26.5, paired *t*-test *P* = .02). Since the distribution of these results was nonparametric, the paired Wilcoxon test was used and showed a median of 6 with anti-EpCAM versus 12 CK-detected cells with the antibody mixture (*P* = .02).

Table 2 shows the additive nature of CTCs captured on the microchannel under different capture and staining conditions. Two tubes of blood were collected from each patient, and capture tested with either anti-EpCAM alone or with the antibody mixture containing anti-EpCAM, unless otherwise specified. The captured cells on the microchannel were initially stained and scored for the presence of CK and CD45, and then stained and scored for CE. The general trend as shown in Table 2 was that an antibody mixture generally captured more classically defined DAPI+/CK+/CD45− cells than anti-EpCAM alone, and that CE revealed more cells than did anti-CK. However, the heterogeneity of cells within samples is apparent even in this small cohort in that the percentage increase in different samples is quite variable. In two of the prostate samples, a third tube of matched blood was obtained and incubated with the same antibody mixture except that anti-EpCAM was omitted. The capture was comparable to the antibody mixture containing anti-EpCAM on prostate samples containing both high and low levels of endogenous CTCs. This suggests that there was an abundance of additional antibodies other than EpCAM bound to the surface of these cells. This is also consistent with increased capture using the antibody mixture since there are CTCs present that do not contain EpCAM (Table 1). The antibody mixture without anti-EpCAM captured more cells than anti-EpCam alone, consistent with the presense of EpCAM-negative cells. 

Table 2 further illustrates that there are EpCAM-positive cells that are captured but lack detectable levels of CK. These cells only become visible when anti-EpCAM-captured cells are stained with CE. In this case, CE is labeling only cells with bound anti-EpCAM since this is the only antibody used for capture. The antibody mixture showed the highest level of CE staining as might be expected. While the antibody mixture showed modestly higher CK-stained cells, significantly more CE cells were detected in all cases, indicating that the antibody mixture was binding to many more CK negative cells than was anti-EpCAM alone. All positive CK or CE cells were CD45 negative. 

**Figure 6:**
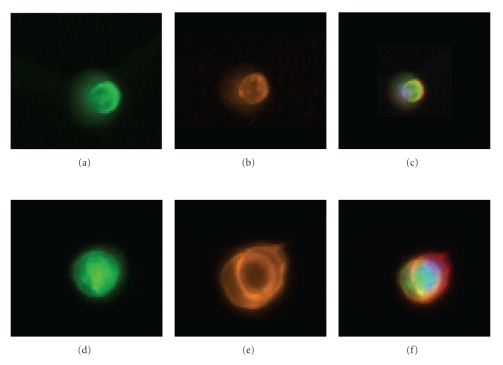
Costaining clinical lung cancer CTCs with anti-CK and CEE-Enhanced. (a–c) A single CTC on the microchannel stained with anti-CK (a), CE-AlexaFluor-546 (orange, (b)), and (c), a composite image. (d–f) shows the same order of staining but with 2 attached CTCs. This demonstrates the costaining of the internal CK antigen and the cell surface antigens with CE.

Dual staining was used to show simultaneous detection using anti-CK and CE. CTCs were captured with the antibody mixture followed by anti-CK (Figures 6(a) and 6(d), labeled with AlexaFluor488, green fluorescence) and then CE (Figures 6(b) and 6(e), labeled with AlexaFluor 546; orange fluorescence). Figures 6(c) and 6(f) are color composite images. Figures 6(d)–6(f) show, an image of two attached CTCs while A–C is a single cell on the microchannel. All relocated DAPI+/CK+/CD45− cells became dual positive for green (CK) and orange (CE).  Figure 6 confirms that CK positive cells are simultaneously labeled with CE. The anti-CK intensity can be augmented with a single color as shown in Figure 5(b) or the CTC can be dual stained with anti-CK and CE having two different fluorescent dyes. 

**Figure 7:**
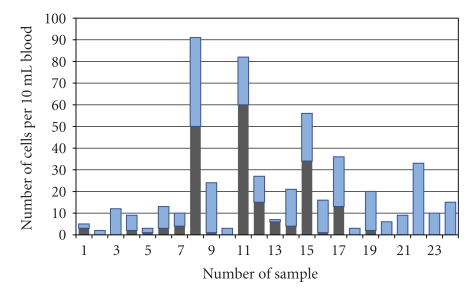
Clinical breast cancer samples sequentially stained with anti-CK and with CEE-Enhanced. The antibody mixture was used to capture CTCs. The dark bars on the bottom represent the number of CK positive cells detected in a sequential series of stage IV breast cancer samples. The location of these cells was recorded and then the channel was restained with CE. The light bars on top represent the newly detected CTCs after CE stain. All cells designated as positive were CD45 negative and DAPI positive.

Figure 7 shows CTCs isolated from a serial set of stage IV breast cancer samples using the antibody mixture for capture. Captured CTCs were first stained with anti-CK, scored, and then stained with CE. The dark bars show the number of CK-positive cells detected and in each case the light bars stacked on top of the dark bars show the additional cells detected with CE. Fifteen of 24 samples (63%) contained CK-positive cells (range 1–60 CTCs) while all of the samples contained at least one additional CE-positive cell (range 1–41; median = 11; Wilcoxon test, *P* = .02). The correlation coefficient (*r* = 0.57, *P* = .004) suggests a weak correlation between CK and CE, but the trend does not suggest that CE is merely a percentage of anti-CK-stained cells. This further suggests that different phenotypic populations of CTC are present within these samples, possibly related to other physiological factors.

## 4. Discussion

In this report, we describe a platform to capture and detect the heterogeneous phenotypes of tumor cells that may exist in patient samples. CTCs were isolated from buffy coats using the EpCAM antibody, or with mixtures of antibodies. Universal detection of specifically captured cells was based on CEE-Enhanced (CE) for *in situ* labeling of the capture antibodies bound to the surface of the captured CTCs within the microchannel. CE costained all CK-positive cells and could be used to enhance the intensity of the CK-stained cells, or to detect cells without CK stain (Figures 5 and 6). Control blood from healthy volunteers showed no positive cells with either anti-CK or CE. 

The use of antibody mixtures and CE showed that additional EpCAM-negative and CK-negative tumor cells were present in peripheral blood. Since both immuno-capture and immuno-detection are antigen-concentration-dependent, the term “negative” here indicates those cells that may contain some antigens, but are below the threshold for either capture or detection employed in this study. Table 2 shows that CTCs captured with anti-EpCAM alone and stained for CK and CE, contained a population of tumor cells that was not CK-positive. Likewise, additional CK positive cells could be identified when a mixture of antibodies was used for capture instead of EpCAM alone. The highest numbers of identified cells were seen when an antibody mixture was used for capture in combination with CE to detect those additional cells that contained very low levels of either EpCAM or CK or both.

The loss of EpCAM or CK in tumor cells has been extensively described. It is not always clear whether the loss of CK is a function of independent oncogenic processes [26–28] or always related to EMT [2, 29]. The present study was focused on detection of CTCs that did not contain either EpCAM or CK. CK can occasionally be aberrantly expressed in lymphocytic cells, though CD45 is used to rule out the CK-positive lymphocytes. Aberrant expression of CK in bone marrow appears to be more common than in peripheral blood [30], and expression caused by inflammatory processes can also contribute to CK false positives [31]. Similar issues surround the upregulation and down-regulation of EpCAM, with similar consequences for the isolation or detection of CTCs in bone marrow or periperal blood. It seems well established that EpCAM is found frequently in tumors [23]; that it can be up-regulated in tumors and has been associated with poor prognosis [24]. From the CTC isolation perspective it is not the finding of CTCs that have expressed EpCAM that is in question, but the concern over false negatives in the failure to detect CTCs that do not express EpCAM. Aside from the fact that not all tumors express EpCAM, there are issues of down-regulation of adhesion molecules in order to metastasize and migrate [25, 32] and the programmed down-regulation of EpCAM as part of EMT [2, 29]. Moreover, EpCAM can be lower as a result of chemotherapy and so CTC enumeration may vary as a result of treatment [33]. 

It is for these reasons that we developed a system to enrich cells with or without EpCAM and integrated this with an *in situ* labeling approach that fluorescently labels those cells with bound capture antibody. Within a biological system containing such a heterogeneous genotypic etiology it may be difficult or impossible to predict with any certainty what kinds of tumor cells might be present in any given sample. If one attempts to apply specificity to the kinds of cells being enriched from a population by using antibodies, it makes sense to visualize the very targets of that enrichment. With heterogeneous samples, one sample may contain mostly EpCAM-positive cells, while the next may have a different phenotype or range of phenotypes. Ancillary tumor-specific markers may then be used to confirm that they are indeed tumor cells or to measure tumor-specific mutations. In this limited study two biopsy-confirmed Her2/neu-positive samples from Figure 7 were found to contain amplified Her2/neu signals in the CTCs that stained with CE-only, in addition to CK-positive cells. Thus CE may be used to identify a wider population of CTCs for further study than would normally be identified with anti-CK alone. 

Isolation of CTCs from metastatic breast cancer samples using an antibody mixture showed additional CE-positive cells and that the numbers were not proportional to the endogenous levels of cells identified by anti-CK staining (Figure 7). While it is desirable to detect more CTCs, the real value of alternate detection using CE may be as much qualitative as quantitative. The proportion of CK-negative or EpCAM-negative CTCs may provide additional diagnostic insights, at very least by suggesting the levels of CTCs that have undergone EMT in a given sample. Specialized antibody mixtures directed to new cancer markers could be developed to enhance the detection of specific populations of CTCs. 

Since the number and type of CTCs is dependent on the isolation and detection technology, it is unlikely that numerical values or cutoffs obtained by CellSearch will be the same for another system. Unlike serum markers, there is no absolute scale for standardization of cell detection across platforms, but rather an individual standard tied directly to a specific enrichment and detection format. There is a wide range of reported values, ranging from numbers roughly comparable to CellSearch to numbers in the hundreds and thousands per mL of blood [6, 10]. Timing is another consideration with regards to enumeration. CTCs isolated as point-of-care within a few hours of blood draw [6, 34] may be the most desirable, though possibly the most challenging approach for widespread use. The half-lives of CTCs in circulation are generally considered to be less than 24 h, possibly only a couple hours [35], though half-lives of days and months have also been reported [36]. The times of collection and storage are significant considerations for enumeration comparisons.

With regards to improved prognostic forecasting it may be of limited value to simply find higher numbers using anti-EpCAM, thus merely resetting the cutoff range of significance. Earlier processing or automated systems with higher sensitivity thresholds resulting in higher CTC values may not necessarily improve prognostic value beyond that achieved by CellSearch. One interesting study in this regard measured all anti-EpCAM and anti-CK-positive “objects” isolated by the CellSearch system [37]. Multiple types of cellular particles and fragments were found to perform equivalently to the classically defined intact CTC in predicting survival. This would suggest that this system would be quite robust at its current level of prognosis, regardless of user bias, since ancillary observations support the same trend. It is in this robustness that the limitations emerge. Systems with associated redundancies reinforce a strong general trend, but by their very nature do not lend themselves to improved specificity. 

Given the heterogeneity of circulating epithelial cells, most of which are assumed to be circulating tumor-associated epithelial cells, the question of detection specificity has not been well studied beyond the parameters of EpCAM and CK. Questions of EMT and the role of these cells at different stages of cancer is of intense interest. Studies in our lab have shown the majority of curable Stages I–III breast cancer samples have significant levels of CE-stained cells but few CK-positive cells (manuscript in preparation). Additional studies are underway.

## 5. Conclusions

The current study demonstrates that the peripheral blood of cancer patients contains circulating tumor cells other than those normally detected with antibodies to EpCAM and cytokeratin. The use of CEE-Enhanced and antibody mixtures along with the traditional anti-EpCAM and anti-CK-based approach may lead to new insights into the diagnostic applications of CTCs.

## Figures and Tables

**Table 1:** Comparison of the number of CK-positive CTCs detected with anti-EpCAM-only and with an antibody mixture (see Section 2).

Tumor type	Anti-EpCAM only	Antibody mix
Breast	0	1
Prostate	37	33
Breast	8	25
Lung	0	0
Breast	8	12
Breast	94	115
Breast	0	1
Prostate	57	97
Prostate	0	0
Colorectal	0	1
Breast	6	16
Lung	1	2
Breast	13	22
Breast	54	72
Breast	0	0
Breast	0	1

**Table 2:** Comparison of the number of CTCs captured using single and multiple antibodies, and detected using anti-CK and CEE-Enhanced.

Cancer type	Anti-EpCAM	Antibody mix	Anti-EpCAM	Antibody mix
	Cytokeratin stain		Additional CTCs detected with CEE-Enhanced stain^b^	
Small Cell Lung Cancer	24	47	+151	+148
Prostate	127	200 (162^a^)	+49	+61
Prostate	2	5 (7^a^)	+11	+27
Prosate	12	10	+2	+25
Colorectal		1		+4

^a^A third tube of matched blood was processed using the antibody mixture that did not contain anti-EpCAM.

^b^All cells were CD45-negative and DAPI-positive.
